# Current status of *Neisseria gonorrhoeae* cervicitis in pregnant women in Japan

**DOI:** 10.1371/journal.pone.0211595

**Published:** 2019-02-07

**Authors:** Shunji Suzuki, Shin-ichi Hoshi, Akihiko Sekizawa, Yoko Sagara, Masanobu Tanaka, Katsuyuki Kinoshita, Tadaichi Kitamura

**Affiliations:** 1 Department of Obstetrics and Gynecology, Japanese Red Cross Katsushika Maternity Hospital, Tokyo, JAPAN; 2 Japan Association of Obstetricians and Gynecologists, Tokyo, JAPAN; 3 Japanese Foundation for Sexual Health Medicine, Tokyo, JAPAN; Emory University School of Medicine, UNITED STATES

## Abstract

We evaluated the current prevalence of gonococcal cervicitis among pregnant women in institutes that either do or do not routinely screen for gonococcal infection in Japan. We requested 2,330 obstetrical facilities to provide information on *Neisseria gonorrhoeae* cervicitis in pregnant women. A total of 1,876 (80.5%) of them responded. The universal screening test for gonococcal cervicitis, involving nucleic acid amplification for all pregnant women, was performed in 281 institutes (13.9% of institutes across Japan). The total rate of pregnant women with gonococcal cervicitis was 1.3% in the institutes performing the screening test during pregnancy, while it was only 0.2% (p < 0.01) in those not performing it. This suggests that 84% of infected women may have been missed in the institutes that do not routinely perform the screening test for gonococcal cervicitis. It may be time to examine the cost-effectiveness of providing gonococcal screening for all pregnant women in Japan.

## Introduction

*Neisseria gonorrhoeae* infection is one of the most common bacterial sexually transmitted diseases (STD). Untreated gonococcal cervicitis in pregnancy has been reported to lead to miscarriage, premature labor associated with chorioamnionitis, and infections of the eyes and pharynx of neonates [1–6]. In 2017, the incidence of gonococcal infection in females was 1.67 per fixed-point medical institution (total of 5,000 institutions) in Japan according to a report on STDs from the Japanese Ministry of Health, Labour and Welfare [7]. Therefore, pregnant women with suspected infection should receive genetic testing for *Neisseria gonorrhoeae* in early pregnancy.

In Japan, all pregnant women can be screened for STDs such as chlamydia cervicitis, syphilis, and human immunodeficiency virus under public insurance coverage, but universal screening for gonococcal cervicitis is not covered. However, the test has been carried out during pregnancy at some obstetric institutes in Japan at pregnant women’s own expense because asymptomatic women with gonococcal cervicitis are not rare [8,9].

Generally, if treated, gonorrhea can be completely eliminated. Based on the guidelines for the office of gynecology in Japan (2017 edition), an intravenous injection of ceftriaxone or cefodizime is recommended for the treatment of gonococcal cervicitis in pregnancy [10]. *Neisseria gonorrhoeae* has developed antimicrobial resistance to various kinds of drugs, even though they are currently recommended as empirical monotherapy against gonorrhea [11–13].

Based on this background, the first aim of this study was to evaluate the current prevalence of gonococcal cervicitis among pregnant women with or without a screening test in Japan. The second aim was to examine the treatment strategy for gonococcal cervicitis during pregnancy in Japan.

## Materials and methods

The protocol for this study was approved by the Ethics Committee of the Japan Association of Obstetricians and Gynecologists (JAOG).

In July 2018, we requested 2,330 obstetrical facilities that are JAOG members to provide information on *Neisseria gonorrhoeae* cervicitis in pregnant women who delivered at ≥ 22 weeks’ gestation between October 1, 2017 and March 31, 2018. A total of 1,876 (80.5%) of the 2,330 obstetrical facilities responded with valid information on a total of 247,212 women, accounting for approximately 53% of all deliveries that occurred in Japan during the study period (approximately 470,000 births in 6 months).

The inquiries in the current study other than those about the prevalence of gonococcal cervicitis were as follows: (1) presence or absence of universal screening test for gonococcal cervicitis involving nucleic acid amplification during pregnancy, (2) reason for the diagnosis of gonococcal cervicitis during pregnancy, (3) name of antibiotics used against gonococcal cervicitis, and (4) number of cases of gonococcal cervicitis with antimicrobial resistance. In this study, unfortunately, we did not inquire about the timing of the screening for gonococcal cervicitis during pregnancy.

The Χ^2^ or Fisher’s exact test was used for categorical variables. Differences with *p* < 0.05 were considered significant.

## Results

Of the 1,876 obstetrical facilities responding with valid information, the screening test for gonococcal cervicitis was performed for all pregnant women at various times during pregnancy in 281 institutes (13.9% of institutes). Table 1 shows the prevalence of the institutes with and without the universal screening test for gonococcal cervicitis during pregnancy in each region of Japan or types of institute. The proportion of screening of gonococcal cervicitis attributable to each region was similar regardless of whether comparing all screening or non-screening clinics or hospitals as shown in Table 1.

**Table 1:** Prevalence of institutes with and without universal screening test for gonococcal cervicitis during pregnancy in each region of Japan or types of institute.

	Universal screening for gonococcal cervicitis	
	Yes	No
Regions in Japan		
Hokkaido-Tohoku	25 (8.9)	149 (9.3)
Kanto	110 (39.1)	550 (34.5)
Chubu-Hokuriku	23 (8.3)	156 (9.8)
Kansai	64 (22.7)	399 (25.0)
Chugoku-Shikoku	14 (5.0)	102 (6.4)
Kyushu	45 (16.0)	239 (15.0)
Types of institute		
Individual clinic	157 (55.9)	854 (53.5)
Hospital	124 (44.1)	741 (46.5)
Total	281 (100)	1,595 (100)

Values are numbers (percentages).

Table 2 shows the distribution of pregnant women with gonococcal cervicitis who delivered at ≥ 22 weeks of gestation in Japan by maternal age in the institutes performing the screening test for gonococcal cervicitis (n = 281 institutes), while Table 3 shows those in the institutes not performing the screening test (n = 1,595 institutes). The total rate of pregnant women diagnosed with gonococcal cervicitis who delivered at ≥ 22 weeks of gestation in Japan was 1.3% (590/46,338) in the institutes performing the screening test for gonococcal cervicitis during pregnancy, while it was 0.2% (421/200,874) in those not performing it. There was a significant difference in the rate between the women with and without the screening test (*p* < 0.01). The rate of gonococcal cervicitis in teenaged pregnant women was higher than that in the other age groups in both institutes with and without performing the screening (*p* < 0.05).

**Table 2:** Distribution of pregnant women with gonococcal cervicitis who delivered at ≥ 22 weeks of gestation in Japan by maternal age in institutes performing the screening test for gonococcal cervicitis (n = 281 institutes).

		*Neisseria gonorrheae* cervicitis					
		Reasons for diagnosis					
Maternal age (y)	Total number	Screening	Awareness of vaginal discharge abnormality	Partner infection history	Incidental diagnosis	Others	Total*
≤ 19	646	13	1	2	3	0	19 (2.9)
20–29	15,550	234	4	1	3	1	243 (1.6)
30–39	27,232	310	0	0	2	0	312 (1.1)
≥ 40	2,910	16	0	0	0	0	16 (0.5)
Total	46,338	573	5	3	8	1	590 (1.3)

*Values are numbers or numbers (percentages).

**Table 3:** Distribution of pregnant women with gonococcal cervicitis who delivered at ≥ 22 weeks of gestation in Japan by maternal age in institutes not performing the screening test for gonococcal cervicitis (n = 1,595 institutes).

		*Neisseria gonorrheae* cervicitis					
		Reasons for diagnosis					
Maternal age (y)	Total number	Screening	Awareness of vaginal discharge abnormality	Partner infection history	Incidental diagnosis	Others	Total*
≤ 19	2,415	-	20	5	0	2	27 (1.1)
20–29	68,657	-	160	25	11	18	214 (0.3)
30–39	117,601	-	130	8	7	23	168 (0.1)
≥ 40	12,201	-	10	0	0	2	12 (0.1)
Total	200,874	-	320	38	18	45	421 (0.2)

*Values are numbers or numbers (percentages).

Of the total of 1,011 women with gonococcal cervicitis, ceftriaxone (intravenous injection), azithromycin (oral), cefodizime (intravenous injection), and other antibiotics were used in 456, 512, 5, and 38, respectively, as the first-line antibiotics. Antimicrobial resistance was only recognized in 3 of the 512 cases using azithromycin (0.6%), while there was no antimicrobial resistance to the other antibiotics used for treatment.

## Discussion

In this study, the rate of pregnant women with gonococcal cervicitis in the institutes performing a screening test for gonococcal cervicitis was significantly higher than in those not performing it. Based on the current results, in institutes without the screening test for gonococcal cervicitis, 84% of infected women may have been missed. Although urethral infections caused by *Neisseria gonorrhoeae* among men can produce symptoms, gonococcal infections in women are commonly asymptomatic or might not produce recognizable symptoms until the development of complications such as pelvic inflammatory disease [8,9,14]. Because cervicitis usually does not cause obvious clinical signs or symptoms, patients may only become aware of the condition after a pelvic examination. When gonococcal infection occurs in neonates, it results from perinatal exposure to the mother’s infected cervix. In these cases, it can manifest as an acute illness such as ophthalmia neonatorum and sepsis leading to arthritis and/or meningitis developing 2–5 days after delivery [4–6,14]. Therefore, prenatal screening and the treatment of pregnant women may be the best methods to prevent gonococcal infection among neonates. The current results may support the recommendation.

In this study, the rate of gonococcal cervicitis in teenaged pregnant women was higher than that in the other age groups, both in institutes that do and do not screen for gonorrhea. The trends are similar to those observed in other STD infections in Japan, such as condylomata acuminate caused by human papillomavirus infection, chlamydia trachomatis and syphilis [15–17]. STD infections in women most commonly occur in young adults [18]. These observations have been suggested to correlate with sexual behavior, showing that younger women have more sexual partners [19], and because the rate of unprotected sex in the younger women has been reported to be high [20].

Many antibiotics that were once effective against *Neisseria gonorrhoeae* including penicillin, tetracycline, and fluoroquinolones are no longer recommended because of high rates of resistance [21]. Oral regimens for the treatment of gonorrhea are limited. For example, resistance to cefixime has reached a level such that it is no longer recommended as a first-line agent in the United States [14]. While cases of resistance to ceftriaxone have been also reported, they are still rare [12,14,21] so the Guidelines for Office Gynecology in Japan (2017 edition) still recommend an intravenous injection of ceftriaxone or cefodizime for the treatment of gonococcal cervicitis (during pregnancy) [10]. Consistent with this, the current study did not reveal any cases of *Neisseria gonorrhoeae* resistant to ceftriaxone or cefodizime, although there were 3 cases (0.6%) resistant to azithromycin. Therefore, the current treatment policy for gonococcal cervicitis in Japan may remain effective.

Unfortunately, we did not examine the neonatal outcomes in the current study; however, it may be time to examine the cost-effectiveness of gonococcal screening for all pregnant women in Japan. In order to improve the perinatal outcomes of gonococcal-infected pregnancy, it also seems appropriate to promote awareness of the necessity of a screening test for gonococcal infection and prevention of STD during pregnancy among Japanese women.
